# Population Pharmacokinetics of Mycophenolic Acid in Renal Transplant Patients: A Comparison of the Early and Stable Posttransplant Stages

**DOI:** 10.3389/fphar.2022.859351

**Published:** 2022-05-09

**Authors:** Peile Wang, Hongchang Xie, Qiwen Zhang, Xueke Tian, Yi Feng, Zifei Qin, Jing Yang, Wenjun Shang, Guiwen Feng, Xiaojian Zhang

**Affiliations:** ^1^ Department of Pharmacy, First Affiliated Hospital of Zhengzhou University, Zhengzhou, China; ^2^ Henan Key Laboratory of Precision Clinical Pharmacy, Zhengzhou University, Zhengzhou, China; ^3^ Department of Kidney Transplantation, First Affiliated Hospital of Zhengzhou University, Zhengzhou, China

**Keywords:** mycophenolic acid, population pharmacokinetics, post-transplant periods, AUC, renal transplantation

## Abstract

Mycophenolic acid (MPA) is an antimetabolic immunosuppressive drug widely used in solid organ transplantation and autoimmune diseases. Pharmacokinetics (PK) of MPA demonstrates high inter- and intra-variability. The aim of this study was to compare the population PK properties of MPA in adult renal transplant patients in the early and stable post-transplant stages and to simulate an optimal dosing regimen for patients at different stages. A total of 51 patients in the early post-transplant period (1 week after surgery) and 48 patients in the stable state (5.5–10 years after surgery) were included in the study. In the two-compartment population PK model, CL/F (23.36 L/h vs. 10.25 L/h) and V/F (78.07 vs. 16.24 L) were significantly different between the two stages. The dose-adjusted area under the concentration time curve (AUC_ss,12h_/dose) for patients in the early stage were significantly lower than those for patients in the stable state (40.83 ± 22.26 mg h/L vs. 77.86 ± 21.34 mg h/L; *p* < 0.001). According to Monte Carlo simulations, patients with 1.0–1.5 g of mycophenolate mofetil twice daily in the early phase and 0.50–0.75 g twice daily in the stable phase had a high probability of achieving an AUC_ss,12h_ of 30–60 mg h/L. In addition, limited sampling strategies showed that two 4-point models (C0-C1-C2-C4 and C1-C2-C3-C6) performed well in predicting MPA exposure by both Bayesian estimate and regression equation and could be applied in clinical practice to assist therapeutic drug monitoring of MPA.

## Introduction

Mycophenolate mofetil (MMF), the morpholino ethyl ester prodrug of mycophenolic acid (MPA), is an immunosuppressive drug widely used in solid organ transplantation and autoimmune diseases (van Sandwijk et al., 2013; Broen and van Laar 2020). After oral administration, MMF is rapidly absorbed from the gastrointestinal tract and converted to MPA, which is then extensively bound to plasma proteins, undergoes extensive hepatic biotransformation to glucuronide metabolites (MPAG and AcMPAG), and is excreted via glomerular filtration (Abd Rahman et al., 2013). Alternatively, the glucuronide metabolites could undergo enterohepatic recirculation (EHC), leading to a secondary peak sometimes observed in the concentration-time profile of MPA (Colom et al., 2014).

The pharmacokinetics (PK) of MPA exhibits significant inter- and intra-individual variability due to factors such as race, gender, albumin level, hepatic and renal function, concomitant medications, gene polymorphism, and other factors that influence the metabolism and excretion of MPA, (van Gelder et al., 2006; Jeong and Kaplan 2007; Tett et al., 2011). These diverse factors have attracted many scholars to perform population PK studies on MPA (Sherwin et al., 2011; Kiang and Ensom 2018; Rong et al., 2019; Reséndiz-Galván et al., 2020; Sheng et al., 2020). Population PK models have become much more comprehensive and refined, incorporating metabolite, free and total MPA, as well as multi-compartmental models capable of describing the EHC process in different populations (Rong et al., 2019; Sheng et al., 2020; Rong et al., 2021), which is important to improve the clinical use of MMF.

Nevertheless, it’s worth noteworthy that previous research and package inserts have shown that MPA exposure was 30–50% lower in the early post-transplant phase than in the stable state when given the same MMF dose (Jeong and Kaplan 2007; Le Meur et al., 2007). However, most studies have focused only on the early stage, the stable stage, or mixed stages (Le Guellec et al., 2004; Veličković-Radovanović et al., 2015). The differences in PK parameters between the early and stable phases were unclear, and the MMF regimen for the stable period was unavailable at present.

Accordingly, the objective of this study was to compare the population PK properties of MPA in adult renal transplant patients in the early and stable state post-transplantation and to provide optimal dosing regimens and estimation approaches for patients at different stages.

## Materials and Methods

### Patients and Study Design

Patients were enrolled at the First Affiliated Hospital of Zhengzhou University during August 2019 and June 2021, and all provided written informed consent. This study was approved by the institutional research ethics committee of First Affiliated Hospital of Zhengzhou University (No. SS-2019–058).

Adult (aged over 18 years) renal transplant patients receiving MMF (MMF dispersible tablets, Hangzhou Zhongmei Huadong Pharmaceutical Co. Ltd., Hangzhou, China) were enrolled. Patients who monitored MPA concentration within 1 week after renal transplantation were assigned to the early post-transplant stage group, and those who monitored MPA concentration during at least 5 years after renal transplantation were assigned to the stable state group. Exclusion criteria were that patients 1) had liver dysfunction before surgery, 2) received a combined organ transplantation, 3) had organ rejection or gastrointestinal disease, and 4) received co-medications that potentially affect MPA PK parameters, such as cholestyramine and rifampicin, according to the drug label. Clinical data, including age, gender, weight, post-transplantation time, medication, tacrolimus concentration, and laboratory results were collected from electronic medical records.

### Mycophenolate Mofetil Administration and Mycophenolic Acid Assay

Patients were received triple immunosuppressive therapy comprising MMF, tacrolimus, and corticosteroids. MMF was administered orally at a dose of 1.0–3.0 g/d (twice daily, bid) in the early stage and 0.5–1.5 g/d in the stable stage under fasting conditions. Triple immunosuppressive dosages for individual depended on the medical teams.

Blood samples were obtained at least 3 days after the same dose. For each patient, one blood sample (2 ml, C0h) was collected immediately at pre-dose, eight to ten blood samples (mainly C0.5h, C1h, C1.5h, C2h, C3h, C4h, C6h, C8h, and C12h) were collected at pre-next dose into EDTA tubes. All samples were centrifuged at 3,500×*g* for 10 min. The supernatant was collected and stored at −80°C until analysis.

MPA plasma concentration was determined by ultra-performance liquid chromatography-diode array detection method as previously described (Zhang et al., 2020). Briefly, 100 μL of plasma sample was mixed with 10 μL of internal standard solution and 200 μL of acetonitrile. After a thorough vortex for 1 min, the mixture was centrifuged at 13,000 rpm for 10 min. Finally, 100 μL of supernatant was transferred to an autosampler vial and 10 μL was injected for quantification. The calibration curves showed an acceptable linearity from 0.04 to 40.0 mg/L, with a lower limit of detection of 0.06 mg/L.

### Non-Compartment Pharmacokinetics Analysis

The PK parameters of MPA were analyzed by non-compartmental analysis (NCA) using Phoenix^®^ WinNonlin software (v8.3, Pharsight, Mountain View, CA, United States). Peak concentration (C_max_) and trough concentration (C_min_) were obtained directly from the raw data. The area under the concentration at steady state for 12 h (AUC_ss,12h_) was calculated by the linear trapezoidal linear interpolation method.

### Population Pharmacokinetics Modeling

The population PK parameters were estimated by the first-order conditional estimation method (FOCE ELS) using Phoenix NLME^®^ software. For the basic modeling, the data were fitted to one or two compartment disposition models with first order absorption kinetics and with or without absorption lag-time (Tlag). The EHC process was also estimated. Parameters for the model included central clearance (CL/F), central distribution volume (V/F), intercompartmental clearance (Q/F), peripheral distribution volume (V2/F), and absorption rate constant (ka). The initial values for these parameters were obtained from NCA results. PK model was selected based on the precision of parameter estimates (standard error), goodness-of-fit plots, and likelihood ratio test (−2LL). Subsequently, inter-individual variability (residual error) was modelled with additive (C_obs_ = C_pred_ + *ε*), proportional [C_obs_ = C_pred_ × (1+ *ε*)] or mixed (additive + proportional) error models, where C_obs_ and C_pred_ were the observed and predicted concentrations, and *ε* was the error variable with a mean of 0 and variance of *σ* ^2^.

For covariate modeling, candidate covariates, including age, sex, body weight, alanine aminotransferase (ALT), aspartate aminotransferase (AST), total bilirubin (TBIL), white blood cells (WBC), erythrocyte, haemoglobin, platelet, serum proteins, albumin, serum creatinine (Scr), creatinine clearance (CrCL), glomerular filtration rate (GFR), urea, and post-transplant stages (the early stage as 0 and the stable stage as 1), evaluated using a stepwise process. Continuous covariates were normalized by median values (of observed values) and categorical covariates were reflected as index variables in the model. By comparison with the initial model, the inclusion criteria for covariates were a drop >6.63 (*p* = 0.01) of objective function value (OFV; −2LL) for forward inclusion and an increase of OFV >10.83 (*p* = 0.001) for backward elimination.

Finally, goodness-of-fit plots were used to evaluate the adequacy of the final model. Model evaluation was carried out using prediction-corrected visual predictive check (pc-VPC) and bootstrap analyses. For pc-VPC, the prediction-corrected observations were plotted against the 5th, 50th, and 95th percentiles of the time-simulated MPA concentrations (*n* = 1,000). A nonparametric bootstrap procedure (*n* = 1,000) was conducted to compare the parameters with the final model parameters estimated from the original data set.

### Monte Carlo Simulations

Based on the final population PK model, six fixed dosing regimens, including 0.25 g bid, 0.50 g bid, 0.75 g bid, 1.0 g bid, 1.25 g bid, and 1.50 g bid in the early and stable stages, were simulated by stochastic simulations (*n* = 1,000) to predict concentrations and AUC_ss,12h_ on day 4. The exposure threshold (AUC_ss,12h_) of MPA was set at 30–60 mg h/L (van Gelder et al., 2006; Jeong and Kaplan 2007; Tett et al., 2011). The dosage regimen with the higher percentage of therapeutic level achieved was selected.

### Limited Sampling Strategies for AUC Estimation

LSSs adopted 3 to 4 timed samples to calculate AUC by both Bayesian and multiple regression analysis. The Bayesian approach was performed on Phoenix^®^ NLME software. Predicted MPA concentrations were estimated from limited sample points based on the final population PK model and then were used to calculate predicted AUC by NCA. Pearson correlation coefficient (r) was used to compare the observed and predicted AUCs. The linear regression analysis was performed on IBM SPSS Statistics (v26.0, SPSS Inc., Chicago, United States). The stepwise forward method was used to deduce the AUC formula based on MPA concentrations at different time points. And then MPA concentrations at different time points were brought into the formula to calculate the predicted AUC. The determination coefficient (r) was used to assess the regression level of the formula.

The predicted AUCs of the two approaches were compared with the observed AUCs. Prediction performance was assessed in terms of accuracy and precision, expressed as prediction error (PE, Eq. 1) and root means square error (RMSE, Eq. 2).

$$\text{PE}\left(\text{\%}\right)=\;\left({\text{AUC}}_{\text{pred}}{-\text{AUC}}_{\text{obs}}\right)\text{×}100/{\text{AUC}}_{\text{obs}}$$ (1)

$$\text{RMSE}=\sqrt{{1/\text{n}\sum \left(\text{PE}\%\right)}^{2}}$$ (2)

## Results

### Patients

The study recruited 51 patients (561 plasma samples) in the early post-transplant stage and 48 patients (518 plasma samples) in the stable state. Blood samples of the two groups were collected on day 4–8 or at least 5 years after renal transplantation, respectively. Table 1 summarized the demographic and clinical information of the patients. There was a significant difference in renal function, concomitant medication dosage, and haemoglobin between the two groups (*p* < 0.001). Patients in early post-transplant stage were on concomitant pantoprazole.

**TABLE 1:** Demographic characteristics of patients.

Characteristic	In the Early Stage (*n* = 51)	In the Stable State (*n* = 48)	*P*
Gender (male, %)	43 (84.31%)	38 (79.17%)	0.507
Age (year)	33.39 ± 8.10	42.29 ± 9.25	<0.001
Weight (kg)	64.02 ± 10.45	65.31 ± 10.54	0.541
Posttransplant time (days)	4.88 ± 1.01	2499.94 ± 467.26	<0.001
MMF dose (mg/day)	1.69 ± 0.44	1.09 ± 0.29	<0.001
Tacrolimus dose (mg/day)	5.97 ± 1.26	1.70 ± 0.43	<0.001
Tacrolimus concentration (mg/L)	11.85 ± 4.71	6.07 ± 1.96	<0.001
Corticosteroid dose (mg/day)			
Methylprednisolone	16.52 ± 4.05	3.89 ± 1.24	<0.001
Prednisone	19.46 ± 3.14	5.0 ± 1.34	<0.001
Serum creatinine (µmol/L)	147.76 ± 73.75	105.27 ± 22.62	<0.001
Creatinine clearance (ml/min)	62.55 ± 20.75	74.51 ± 19.29	0.004
GFR (mL/min⋅1.73m^2^)	61.26 ± 25.02	74.17 ± 15.93	0.003
Uric acid (mmol/L)	272.10 ± 109.77	373.29 ± 88.97	<0.001
Urea (mmol/L)	13.09 ± 5.81	7.28 ± 2.02	<0.001
Total protein (g/L)	61.58 ± 5.46	69.23 ± 5.55	<0.001
Serum albumin (g/L)	41.94 ± 4.95	46.07 ± 2.24	0.001
ALT (U/L)	13.20 ± 7.95	12.19 ± 7.36	0.514
AST (U/L)	11.33 ± 4.79	12.66 ± 5.64	0.671
Total bilirubin (µmol/L)	11.14 ± 3.79	10.93 ± 6.59	0.845
Erythrocyte (10^12^/L)	3.22 ± 0.54	4.56 ± 0.57	<0.001
Haemoglobin (g/L)	99.43 ± 16.09	139.81 ± 18.23	<0.001
White blood cells (10^9^/L)	6.74 ± 2.49	7.06 ± 1.97	0.485
Platelet (10^9^/L)	131.69 ± 37.88	204.50 ± 50.61	<0.001

MMF, mycophenolate mofetil; GFR, glomerular filtration rate; ALT, alanine aminotransferase; AST, aspartate aminotransferase; values were mean ± standard deviation or No. (%).

### Pharmacokinetics Profiles

**FIGURE 1:**
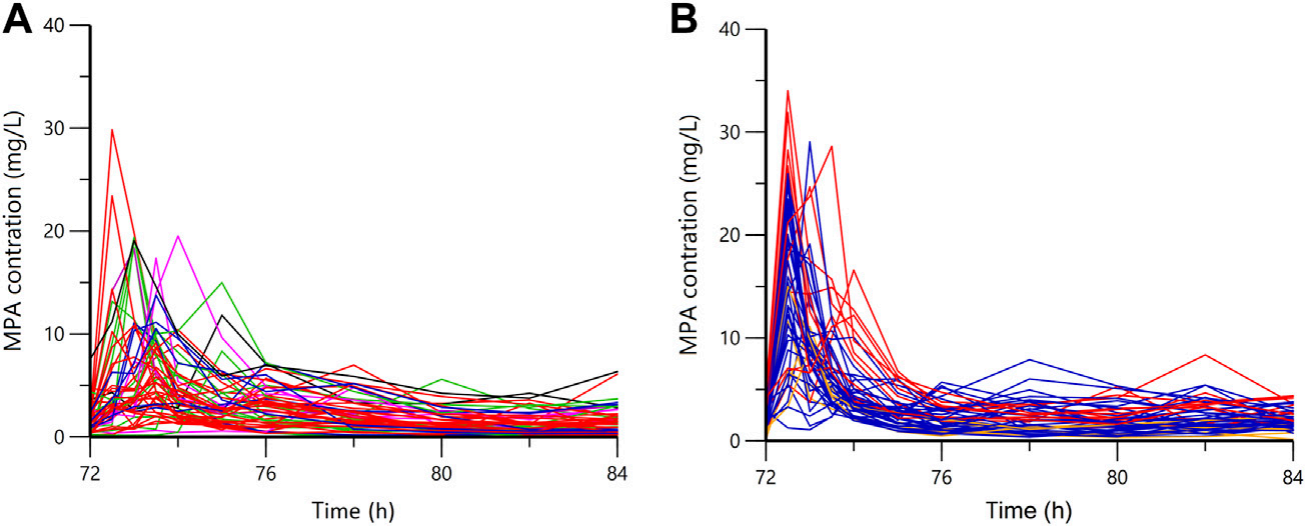
The plasma concentration-time profiles of mycophenolic acid. **(A)** in the early stage; **(B)** in the stable state; orange, 0.25 g bid; blue, 0.5 g bid; red, 0.75 g bid; green, 1.0 g bid; purple, 1.25 g bid; black, 1.5 g bid.

**FIGURE 2:**
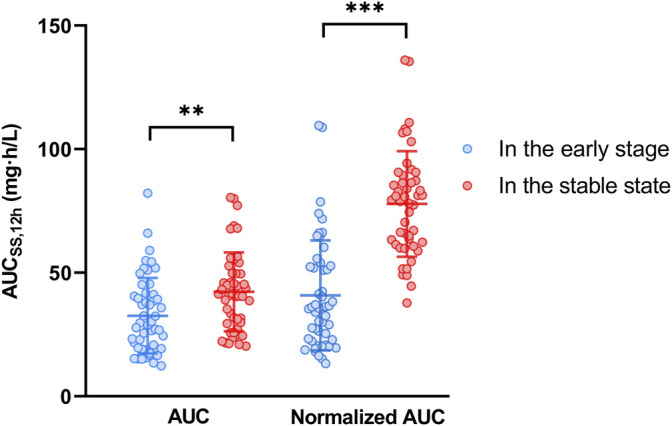
The mycophenolic acid exposures in the early and stable post-transplant stages. Normalized AUC_ss,12h_ was adjusted to the dose/AUC_ss,12h_; ** represented *p* < 0.01; *** represented *p* < 0.001.

Plasma concentration time profiles and PK parameters were shown in Figure 1 and Table 2, respectively. PK parameters were significantly different between the two groups (*p* = 0.05). Preliminary MPA concentration showed large individual variability, ranging from below the quantification limit (0.1 mg/L) to 34.0 mg/L. Delayed absorption at 2–4 h post-dosing and EHC at 6–12 h post-dosing were observed in some patients. The AUC reached the target exposure in 51 patients (22 in the early stage and 29 in the stable stage). After adjusted AUC with dosage (AUC/dose), the mean normalized AUC_ss,12h_ of MPA (40.83 ± 22.26 mg h/L) in the early post-transplant period group was significantly lower than that (77.86 ± 21.34 mg h/L; *p* < 0.001) in the stable post-transplant period group (Figure 2).

**TABLE 2:** Summary of the pharmacokinetic parameters of mycophenolic acid.

Variables	In the Early Stage	In the Stable State	*P*
AUC_ss,12h_ (mg·h/L)	32.61 ± 15.32	42.30 ± 15.94	0.003
AUC_ss,12h_/dose	40.83 ± 22.26	77.86 ± 21.40	<0.001
CL/F (L/h)	24.21 ± 15.23	8.66 ± 3.52	<0.001
Vd/F (L)	165.35 ± 109.34	97.56 ± 56.66	<0.001
C_min_ (mg/L)	0.96 ± 0.88	1.29 ± 0.75	0.046
C_max_ (mg/L)	9.83 ± 6.04	16.87 ± 7.49	<0.001
T_max_ (h)	74.10 ± 1.54	72.82 ± 0.58	<0.001
t_1/2_ (h)	6.10 ± 4.67	11.99 ± 17.54	0.030

### Population Pharmacokinetics Models

The raw concentration data were adequately described by a two-compartmental model with first order absorption with lag-time and linear elimination. Inter-individual variability was described by the proportional error model. Additionally, the inclusion of the EHC process did not significantly decrease OFV value (ΔOFV = 7.58; Supplementary Table S1 and Supplementary Figure S1). In the covariate analysis, post-transplant stages were the covariate for V (ΔOFV = 70.72, *p* < 0.001) and CL (ΔOFV = 57.41, *p* < 0.001). Other covariates, including age, gender, body weight, ALT, AST, WBC, haemoglobin, serum proteins, albumin, TBIL, Scr, CrCL, GFR, and urea, showed no significant effect on PK parameters (Supplementary Figure S2). Finally, the final population PK model parameters were shown in Table 3. Typical PK parameters for patients in the early post-transplant stage, including CL/F (23.36 L/h vs. 10.25 L/h) and V/F (78.07 vs. 16.24 L), were considerably different from the values for patients in the stable state.

**TABLE 3:** Parameter estimates of the final population pharmacokinetic model.

Parameter	Final Model				Bootstrap		
	Estimate	CV (%)	95% CI	Shrinkage (%)	Median	CV (%)	95% CI
tvka (1/h)	1.36	10.30	1.08–1.63	NA	1.56	20.70	1.11–2.36
tvV/F (L)	78.07	12.95	58.23–97.90	NA	88.53	41.32	54.26–132.43
tvV2/F (L)	554.52	18.85	349.31–759.34	NA	561.53	48.88	176.51–1,229.76
tvCL/F (L/h)	23.36	6.40	20.42–26.29	NA	19.69	19.94	11.48–24.73
tvQ/F (L/h)	29.53	9.72	23.90–35.17	NA	36.46	36.49	23.02–56.0
tvTlag (h)	0.23	10.40	0.19–0.28	NA	0.26	14.06	0.21–0.35
dVdStage	−2.54	−15.32	−3.29–1.77	NA	−2.36	−39.68	−3.36–0.18
dCLdStage	−0.82	−10.01	−0.98–0.66	NA	−0.80	−22.72	−1.09–0.36
Inter-individual variability							
*ω* ^2^V	1.03	38.83	0.25–1.81	21.47	1.29	31.01	0.51–2.07
*ω* ^2^CL	0.20	60.0	0.04–0.44	14.24	0.19	63.16	0.05–0.53
*ω* ^2^Ka	0.34	58.82	0.05–0.73	13.97	0.40	50.0	0.01–0.79
*ω* ^2^V2	1.72	38.95	0.41–3.03	16.33	1.71	39.18	0.40–3.02
*ω* ^2^Q	0.98	35.71	0.29–1.67	11.34	0.87	40.23	0.18–1.56
*ω* ^2^Tlag	0.74	20.27	0.45–1.03	28.37	0.81	18.52	0.52–1.10
Residual variability (*σ*)							
stdev0	0.37	4.77	0.33–0.40	4.41	0.39	6.15	0.33–0.42

SE, standard error; CV, percent confidence of variation; CI, confidence interval; tvka, typical value of absorption rate constant (ka); V, central compartment distribution volume; V2, peripheral compartment distribution volume; CL, central compartment clearance; Q, inter-compartmental clearance (CL2); Tlag, lag time of first-order absorption; dCLdStage, fixed parameter coefficient of CL, to post-transplant stage; stdev0, standard deviation; NA, not applicable.

**FIGURE 3:**
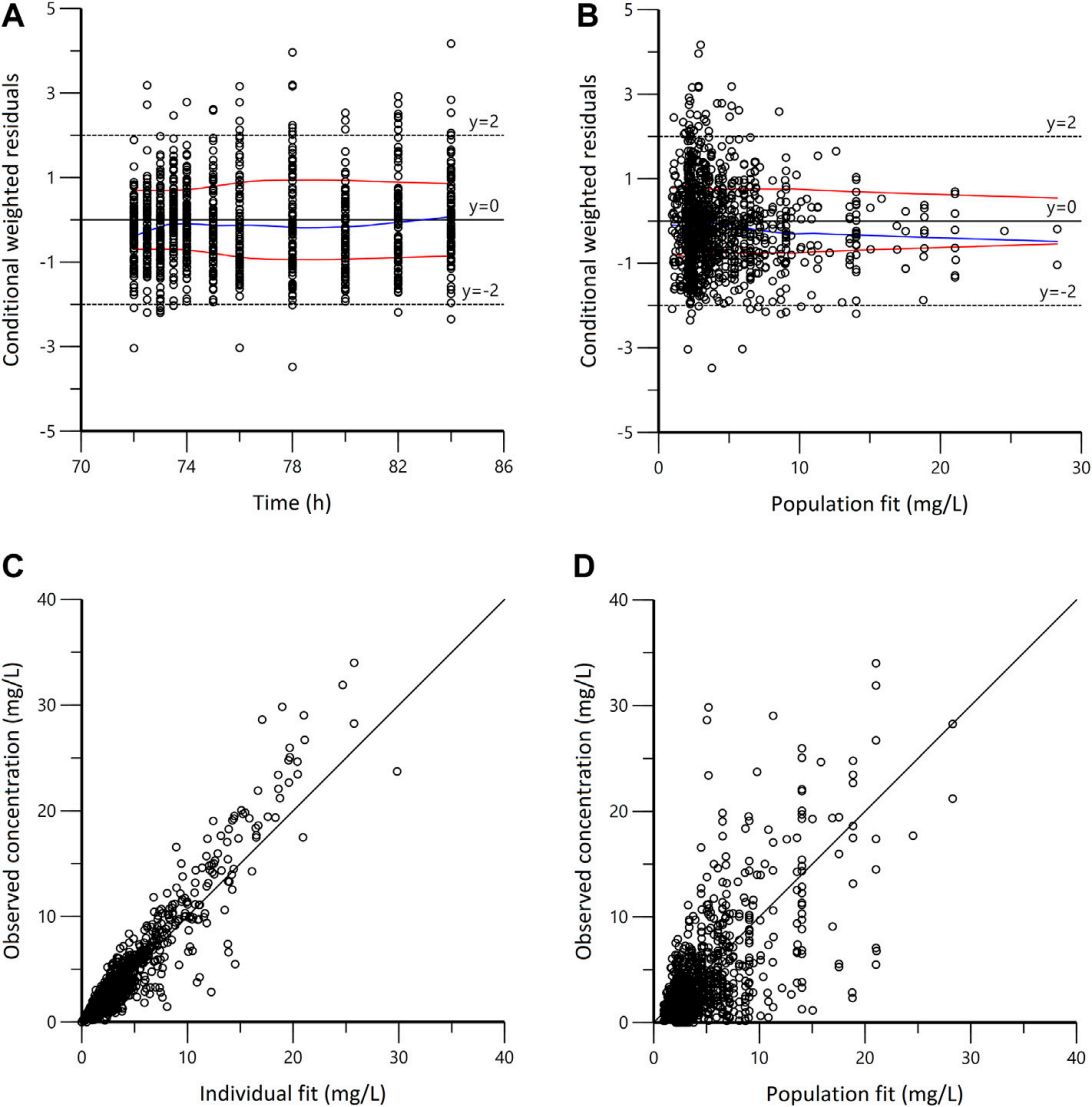
Goodness-of-fit plots for the final population pharmacokinetic model. **(A)** Conditional weighted residuals vs. time (CWRES vs. IVAR); **(B)** Conditional weighted residuals versus population fit (CWRES vs. PRED); **(C)** Observed versus individual fit (DV vs. IPRED); **(D)** Observed versus population fit (DV vs. PRED). The blue lines in panels A and B represent smoothed regression lines; the red lines represent compute absolute regression lines (with its negative reflection).

**FIGURE 4:**
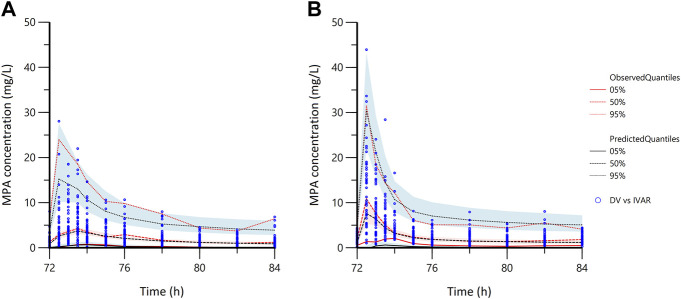
The prediction corrected-visual predictive check of the population pharmacokinetic models. **(A)** in the early stage; **(B)** in the stable stage; the blue dots represent the prediction corrected observed data; DV, observed data; IVAR, time since the last dose.

In the goodness-of-fit plots (Figure 3), the plots were normally distributed with no obvious systematic bias. In the pc-VPC plots (Figure 4), most of observed data were within the 5th-95th prediction percentile. Consistently, the bootstrap results closely matched the mean estimates from the population PK models, confirming the stability of the model.

### Model-Based Simulations

**FIGURE 5:**
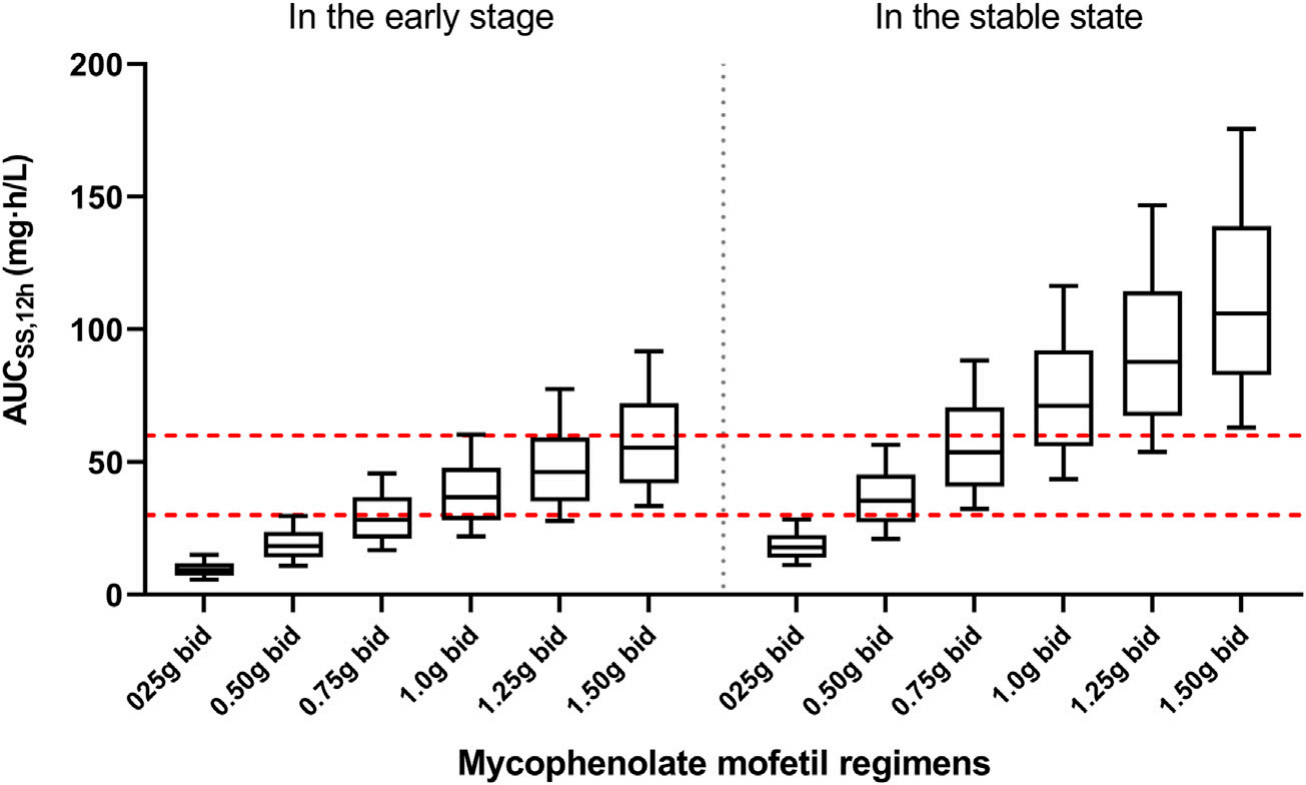
The plot present simulation results of mycophenolic acid exposures. The red dot lines present the upper and lower limit of targeted exposure (30–60 mg h/L). The black solid lines present the median and 10th-90th percentile of simulated exposure.

Figure 5 presented the AUC_ss,12h_ for various MPA dosages. In the early post-transplant stage, the probability of achieving target exposure (30–60 mg h/L) was 0.1, 9.0, 41.2, 59.4, 62.0, and 51.2% for six fixed regimens (0.25, 0.50, 0.75, 1.0, 1.25, and 1.50 g bid), respectively. As for the stable post-transplant period, the likelihood of achieving efficacious exposure for six fixed regimens (0.25, 0.50, 0.75, 1.0, 1.25, and 1.50 g bid) were 7.3, 60.1, 53.3, 31.7, 15.0, and 7.5%, respectively.

### LSSs for AUC Estimation

Based on the literature (Filler 2004; Kiang and Ensom 2016), seven 3 or 4-point samples models were selected, while the whole-point model was provided as a reference. The predictive performance of Bayesian and multiple regression analyses was presented in Table 4, and the regression equations were shown in Supplementary Table S2. For both analyses, the predictive ability of 4-time point schemes was slightly higher than that of 3-time point schemes, but the improvement of models was limited. Two strategies (C0-C1-C2-C4 and C1-C2-C3-C6) showed good correlations with the observed AUC in the early staged and stable stage, respectively.

**TABLE 4:** Limited sampling strategies for AUC_ss,12h_ estimation.

Time Points	Bayesian Approach			Multiple Regression Analysis		
	r	PE (%)	RMSE (%)	r	PE (%)	RMSE (%)
In the early stage						
1, 4, 6	0.86	2.34 ± 24.19	3.36 ± 4.56	0.88	4.66 ± 24.31	3.43 ± 4.38
0, 1, 4	0.87	2.09 ± 23.79	3.31 ± 4.24	0.91	4.24 ± 21.40	3.03 ± 3.56
0, 1, 8	0.77	5.35 ± 32.75	4.97 ± 6.77	0.90	4.99 ± 23.55	3.34 ± 4.71
1, 2, 4	0.87	-4.99 ± 22.10	3.14 ± 3.97	0.87	4.53 ± 25.79	3.63 ± 4.49
0, 1, 2, 4	0.90	-3.42 ± 19.41	2.73 ± 3.40	0.93	3.30 ± 19.40	2.73 ± 3.09
1, 1.5, 2, 4	0.84	-2.43 ± 23.22	3.23 ± 5.19	0.88	4.49 ± 25.54	3.60 ± 4.32
1, 2, 3, 6	0.88	-5.28 ± 20.14	2.88 ± 4.07	0.93	3.20 ± 19.24	2.70 ± 3.77
All points	0.97	0.13 ± 8.23	1.17 ± 1.52	1.0	-0.02 ± 2.03	0.28 ± 0.54
In the stable state						
1, 4, 6	0.87	-8.57 ± 19.22	2.75 ± 3.87	0.84	4.39 ± 20.94	3.06 ± 4.66
0, 1, 4	0.85	-5.83 ± 19.79	2.67 ± 4.01	0.82	5.02 ± 22.44	3.29 ± 5.99
0, 1, 8	0.89	-6.42 ± 18.89	2.58 ± 3.07	0.89	3.24 ± 18.92	2.80 ± 3.09
1, 2, 4	0.83	-10.0 ± 21.20	3.10 ± 4.29	0.84	4.85 ± 21.86	3.34 ± 4.45
0, 1, 2, 4	0.86	-3.72 ± 20.84	2.74 ± 4.33	0.88	3.90 ± 19.33	2.82 ± 3.51
1, 1.5, 2, 4	0.83	-10.4 ± 21.25	3.13 ± 5.28	0.87	4.59 ± 20.70	3.17 ± 4.28
1, 2, 3, 6	0.91	-10.9 ± 14.78	2.63 ± 2.93	0.90	3.28 ± 17.46	2.82 ± 2.99
All points	0.98	-3.89 ± 7.29	1.17 ± 1.23	1.0	0.07 ± 0.45	0.07 ± 0.17

AUC_ss, 12h_, the area under the concentration across 12 h at steady state; PE, prediction error; RMSE, root mean square error.

## Discussion

Due to the inter- and intra-individual variability, a low probability of reaching the target (51.5%) was observed (Figure 2). The individualization of MMF dosage and time-dependent clearance of MPA in renal transplant recipients have been well recognized. In the present study, PK characteristics of MPA in renal transplant recipients co-treated with tacrolimus and corticosteroids in the early and stable stages were analyzed and compared. Similar to previously published models (Sherwin et al., 2011; Kiang and Ensom 2018), a two-compartment with first-order absorption with lag time and linear elimination model best described the MPA data. The population PK parameter (Table 3) were in line with those reported in other studies (ka = 0.64–4.0 h^−1^, V/F = 10.3–75.9 L, V2/F = 183–4910 L, CL/F = 11.8–26.3 L/h, Q/F = 11.2–38.0 L/h, and Tlag = 0.16–0.57 h) (Cremers et al., 2005; de Winter et al., 2010; Lamba et al., 2010; de Winter et al., 2011; de Winter et al., 2012; Rong et al., 2019; Sheng et al., 2020).

However, in the present study, the CL/F and V/F values were substantially different between the early and stable post-transplant periods. In particular, the CL/F for patients in the early period was almost twice as high as that in the stable state (23.36 L/h vs. 10.25 L/h). This was in agreement with the fact that MPA exposure in the early post transplantation period was approximately 30–50% lower for the same dose than in the late post transplantation period (Jeong and Kaplan 2007). This was also confirmed by a comparison of the dose-adjusted AUC_ss,12h_ between the two groups (40.83 ± 22.26 mg h/L vs. 77.86 ± 21.40 mg h/L; *p* < 0.001; Table 2).

Due to the EHC process, few population PK analyses have used physiological or mechanism-based models to describe the EHC of MPA (Sherwin et al., 2011). In previous studies, the contribution of EHC to MPA exposure ranged from 10 to 61% (mean 37%) in healthy individuals. However, a growing number of studies have shown an insignificant effect of EHC on MPA exposure in Asian participants receiving MMF and prednisolone (4.4–10.9%) (Yau et al., 2009; Sheng et al., 2020).

In several published population PK models, age, body weight, albumin, CrCL, post-transplant time, and gene polymorphisms have been identified as covariates for MPA PK parameters (Atcheson et al., 2004; van Hest et al., 2007; Veličković-Radovanović et al., 2015; Kiang and Ensom 2018; Sheng et al., 2020). However, different covariates have been found in different literature, and post-transplant time was always a covariate affecting the PK parameters of MPA. In the present study, no effect of age, body weight, or albumin on PK parameters was observed (Supplementary Figure S2). This could be explained by the relatively small variation in these factors, with the 10th-90th percentiles of 26–50 years, 52.0–78.0 kg, and 37.8–48.5 g/L, respectively. Although there might be a correlation between CrCL and CL (Supplementary Figure S2), the effect of CrCL on PK parameters was too small to be included in the model compared to post-transplant stages. Regarding gene polymorphisms, our previous study found that the AUC/dose of MPA in 233 adult renal transplant patients was only marginally associated with the polymorphism of *UGT2B7* 802C > T (*p* = 0.021) and *SLCO1B1* 521T > C (*p* = 0.036) (Wang et al., 2020); thus, gene polymorphisms was not included in this study.

In addition to the factors above, erythrocyte, haemoglobin, and the dosage of concomitant medication were significant differences between the two groups (*p* < 0.001, Table 1). As for erythrocyte and haemoglobin, an *in vitro* study discovered that the percentage of MPA in erythrocytes was very small (0.001%), making this reason impossible (van Hest et al., 2007). In terms of concomitant medication, the significant effect of corticosteroids on MPA clearance was found in previous studies (Cattaneo et al., 2002). Rong *et al.* reported that renal transplant patients receiving corticosteroid-free regimen had significantly lower MPA clearance (CL/F = 2.87 L/h) and that MPA clearance was positively correlated with AUC_MPAG_/AUC_MPA_. The mechanism was most likely mediated by a change in MPA hepatic intrinsic clearance; specifically, the inducement of UGT enzymes involved in MPA glucuronidation (Rong et al., 2019). In clinical practice, 500–1,000 mg (10–15 mg/kg) methylprednisolone was regular given intravenously during transplantation and 250–500 mg intravenously during the first 3 days after surgery. From the fourth day to 1 month after surgery, the corticosteroid dosage was reduced to 10–30 mg/d orally, and then to 5.0–7.5 mg/d orally after 6 months (Organ Transplantation Society of Chinese Medical Association 2019). High corticosteroid dosages within the first week may activate UGT enzymes activity, resulting in increased MPAG concentration and MPA clearance.

In addition, coadministration of proton pump inhibitors decreases the extent of MPA absorption by approximately 20% (Rissling et al., 2015; Sunderland et al., 2020). Since all patients were taking pantoprazole concomitantly in the first week post-transplant, which would also be an important factor affecting MPA PK parameters. Given that, age, body weight, albumin, haemoglobin, and gene polymorphisms were not associated with changes in CL/F. Coadministration of corticosteroid and proton pump inhibitors may be the major reason for the lower CL/F of MPA in the stable stage compared to that in the early state.

Based on the Monte Carlo simulations (Figure 5), the median AUC_ss,12h_ for patients in the early post-transplant period was much lower than that in the stable post-transplant period at the same dose. With an exposure threshold of 30–60 mg h/L, 1.0–1.5 mg twice daily for patients in the early posttransplant period and 0.50–0.75 mg twice daily for patients in stable post-transplant period would be the optimal regimens.

Because obtaining multiple samples throughout a dosing interval to estimate AUC is not always feasible in clinical practice, LSSs are typically used to predict drug exposure using Bayesian estimates and regression equations. In general, Bayesian analysis has excellent prediction performance and sample timing flexibility, but it requires sophisticated software with sufficient prior population PK data; whereas regression analysis has inferior predictive performance and sample timing flexibility, but it is computationally simple (Kiang and Ensom 2016). In the present study, the accuracy and precision of two approaches were comparable for either 3 or 4-point models. Both methods revealed good correlation between AUC_ss,12h_ and two four-point models (C0-C1-C2-C4 and C1-C2-C3-C6), which was consistent with the literature and could be used for MPA TDM (Filler 2004; Pawinski et al., 2013).

There are several limitations. Firstly, due to limited sample size, the population PK models and LSSs were not externally validated in a separate cohort. Second, only patients in the first week or 5 years after transplantation were enrolled in order to accurately estimate PK parameters for patients in the two stages. PK parameters for other stages were not available. Third, free MPA concentrations were not determined. Atcheson *et al.* recommended that free instead of total MPA concentrations should be monitored clinically when plasma albumin was ≤31 g/L (Atcheson et al., 2004); however, the albumin values in this study were all ≥33.7 g/L. Finally, given our hypothesis that MPAG is an important factor influencing MPA clearance, more research should be conducted to determine MPAG concentrations and assess the effect of corticosteroid dosage on UGT enzymes. Despite these limitations, this is the first study that provided the reference dosages of MMF for renal transplant patients at different stages and validated the limited sampling methods for AUC estimation.

## Conclusion

In conclusion, the two-compartment population PK model showed that CL/F of MPA in the early post-transplant stage was much lower than that in the stable state (23.36 L/h vs. 10.25 L/h). Accordingly, 1.0–1.5 g of MMF twice daily for patients in the early period and 0.50–0.75 g twice daily for patients in the stable period would be the optimal regimens to achieve the target AUC_ss,12h_. Furthermore, LSSs suggested two 4-point models (C0-C1-C2-C4 and C1-C2-C3-C6) performed well in predicting MPA exposure using Bayesian estimate and regression equation and could be applied in clinical practice to assist TDM of MPA.

## Data Availability

The raw data supporting the conclusions of this article will be made available by the authors, without undue reservation.
