# Supplementary material for: Population Pharmacokinetics of Mycophenolic Acid in Renal Transplant Patients: A Comparison of the Early and Stable Posttransplant Stages
Source: Front Pharmacol. 2022 May 9;13:859351. doi: 10.3389/fphar.2022.859351 (PMC9126255; doi:10.3389/fphar.2022.859351)
Supplement: Supplementary file 1 [file DataSheet1.docx]

**Table S1** Population pharmacokinetic model building process (n = 99).

| Model | Structural model description | Error model | OFV |
| --- | --- | --- | --- |
| 1 | One-compartment model, first-order absorption, linear elimination | Additive | 5242.47 |
| 2 | One-compartment model, first-order absorption, linear elimination | Multiplicative | 4687.21 |
| 3 | One-compartment model, first-order absorption, linear elimination | Additive+ Multiplicative | 4737.77 |
| 4 | Two-compartment model, first-order absorption, linear elimination | Additive | 4968.33 |
| 5 | Two-compartment model, first-order absorption, linear elimination | Additive+ Multiplicative | 4131.93 |
| 6 | Two-compartment model, first-order absorption, linear elimination | Multiplicative | 4082.96 |
| 7 | Two-compartment model, first-order absorption, linear elimination, EHC | Multiplicative | 4075.48 |
| 8 | Two-compartment model, first-order absorption with lag time, linear elimination | Multiplicative | 4057.31 |
| 9 | Two-compartment model, first-order absorption, linear elimination with lag time, V-stage | Multiplicative | 3986.59 |
| 10 | Two-compartment model, first-order absorption, linear elimination with lag time, V-stage and CL-stage | Multiplicative | 3929.18 |

OFV, objective function value; EHC, enterohepatic recirculation; V, central compartment distribution volume; CL, central compartment clearance; stage, post-transplant stages (the early stage as 0 and the stable stage as 1).

**Table S2** The regression equations for AUC_ss,12h_ estimation.

| Time points | Equations |
| --- | --- |
| In the early stage | |
| 1, 4, 6 | 4.251+1.137×C1+4.489×C4+3.088×C6 |
| 0, 1, 4 | 8.288+4.767×C0+0.624×C1+4.082×C4 |
| 0, 1, 8 | 12.042+5.080×C0+0.382×C1+6.419×C8 |
| 1, 2, 4 | 5.850+0.878×C1+1.266×C2+4.975×C4 |
| 0, 1, 2, 4 | 6.835+4.195×C0+0.571×C1+0.935×C2+3.593×C4 |
| 1, 1.5, 2, 4 | 5.540+0.803×C1+0.296×C1.5+1.047×C2+4.955×C4 |
| 1, 2, 3, 6 | 1.522+1.123×C1+1.211×C2+1.967×C3+5.026×C6 |
| In the stable state | |
| 1, 4, 6 | 13.237+0.929×C1+4.212×C4+4.388×C6 |
| 0, 1, 4 | 10.565+5.581×C0+0.920×C1+4.685×C4 |
| 0, 1, 8 | 8.180+3.338×C0+1.273×C1+7.072×C8 |
| 1, 2, 4 | 11.979+1.002×C1+2.160×C2+4.092×C4 |
| 0, 1, 2, 4 | 7.599+4.430×C0+0.890×C1+1.875×C2+3.078×C4 |
| 1, 1.5, 2, 4 | 14.155+0.471×C1+1.389×C1.5+1.142×C2+3.447×C4 |
| 1, 2, 3, 6 | 8.810+0.998×C1+1.589×C2+1.746×C3+4.495×C6 |


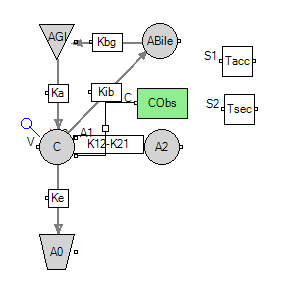


**Figure S1** The graphical template for enterohepatic recirculation. AGI, Intestinal absorption compartment; ABile, Bile accumulation compartment; ka, absorption rate constant; ke, elimination rate constant; Tacc, accumulation time; Tsec, excretion time.


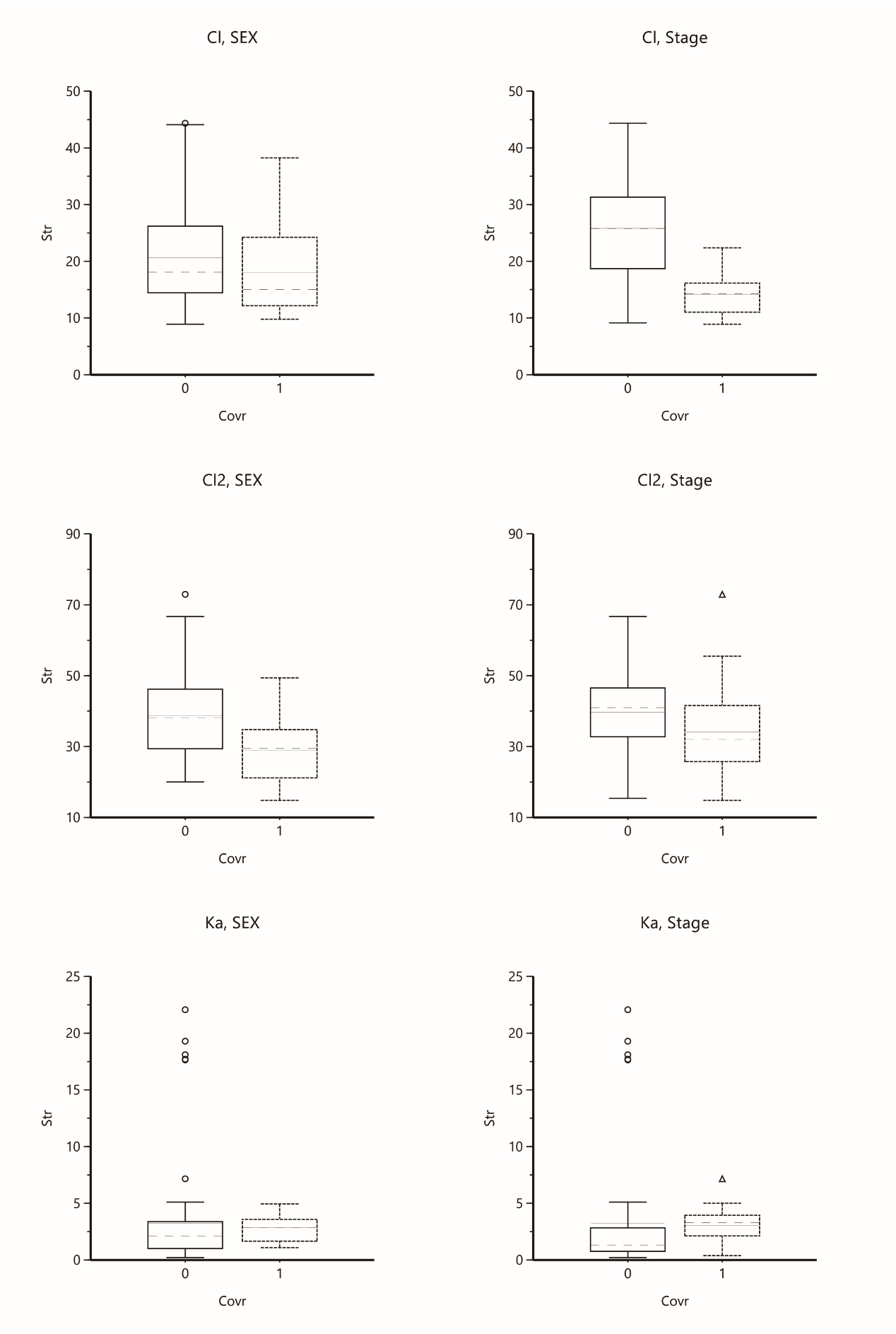


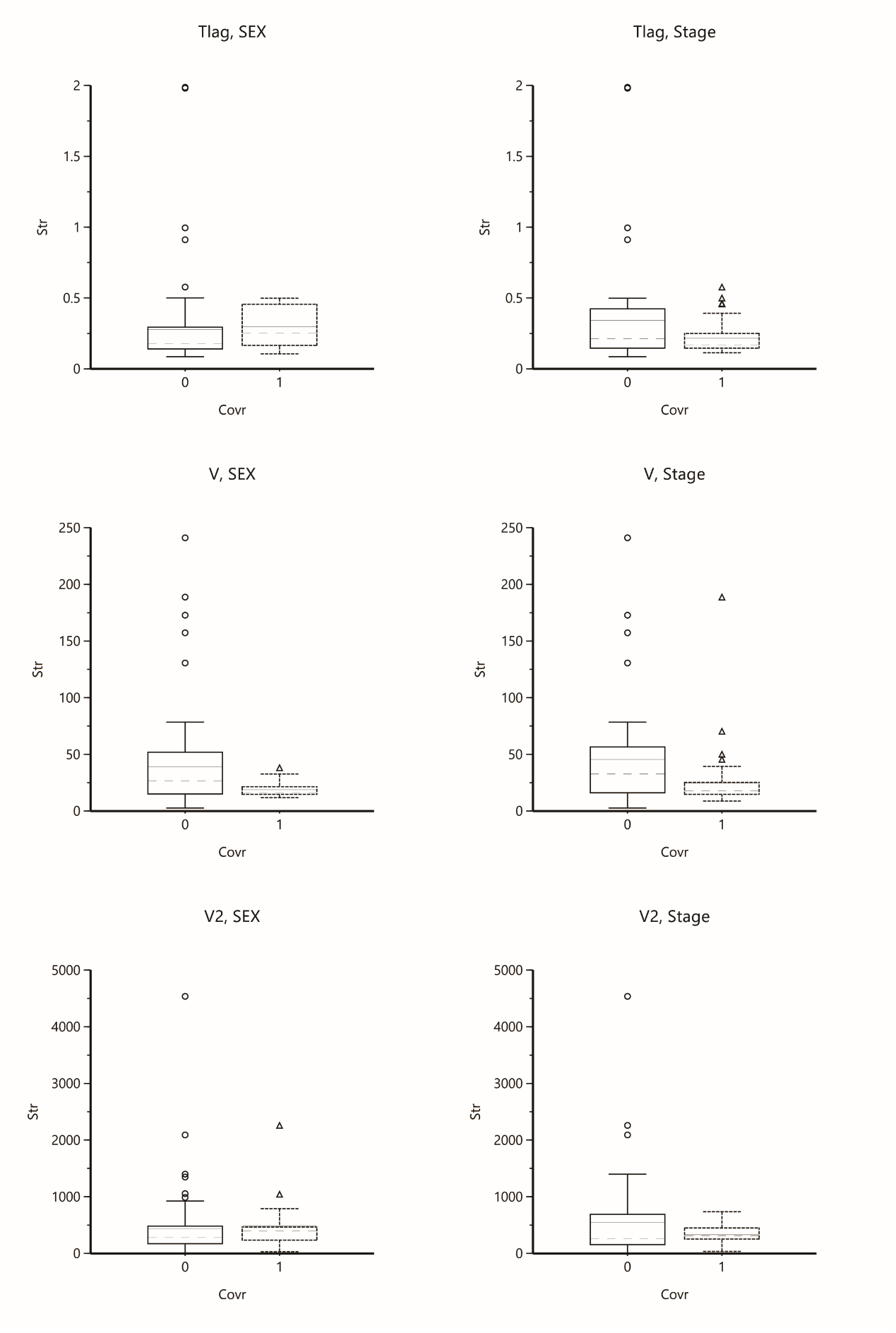


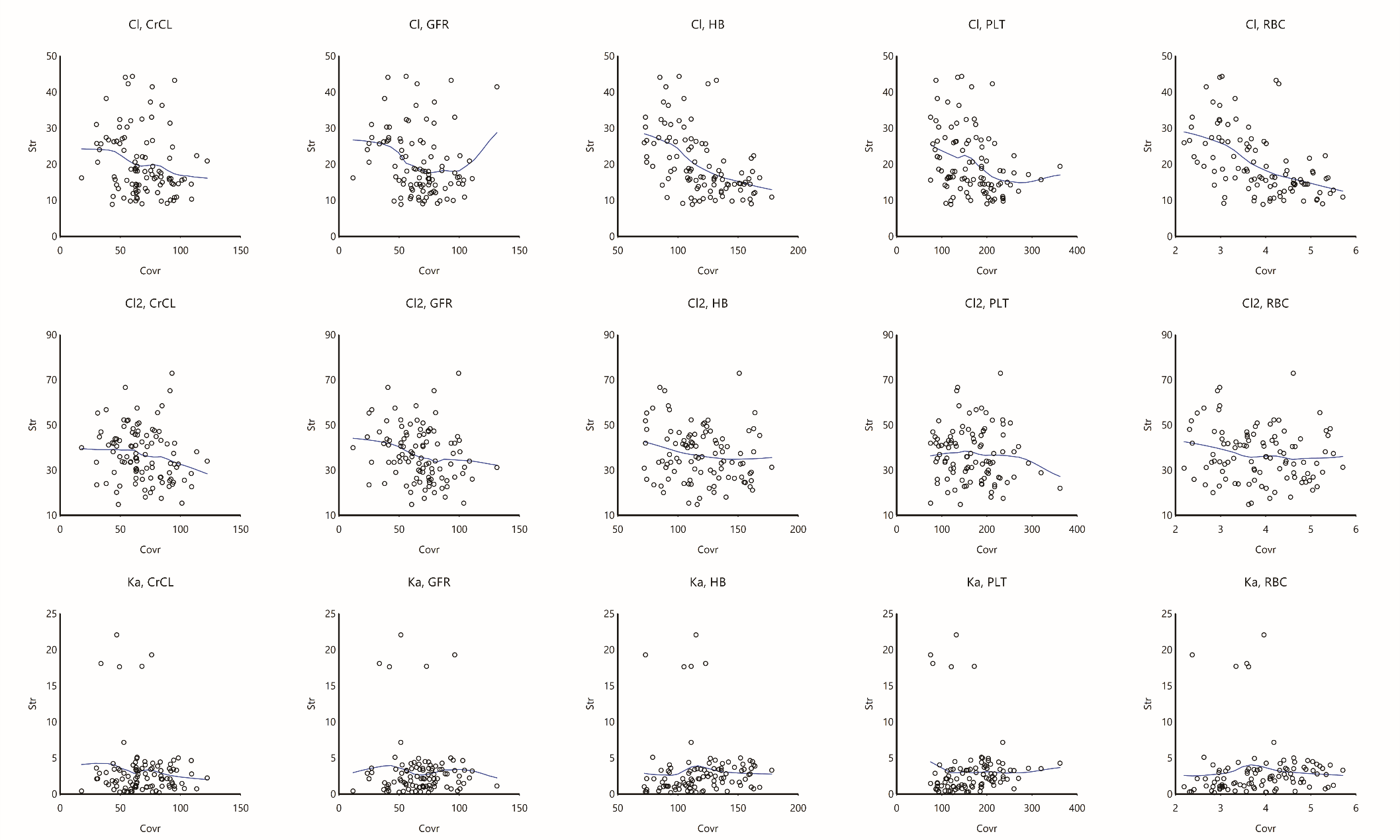

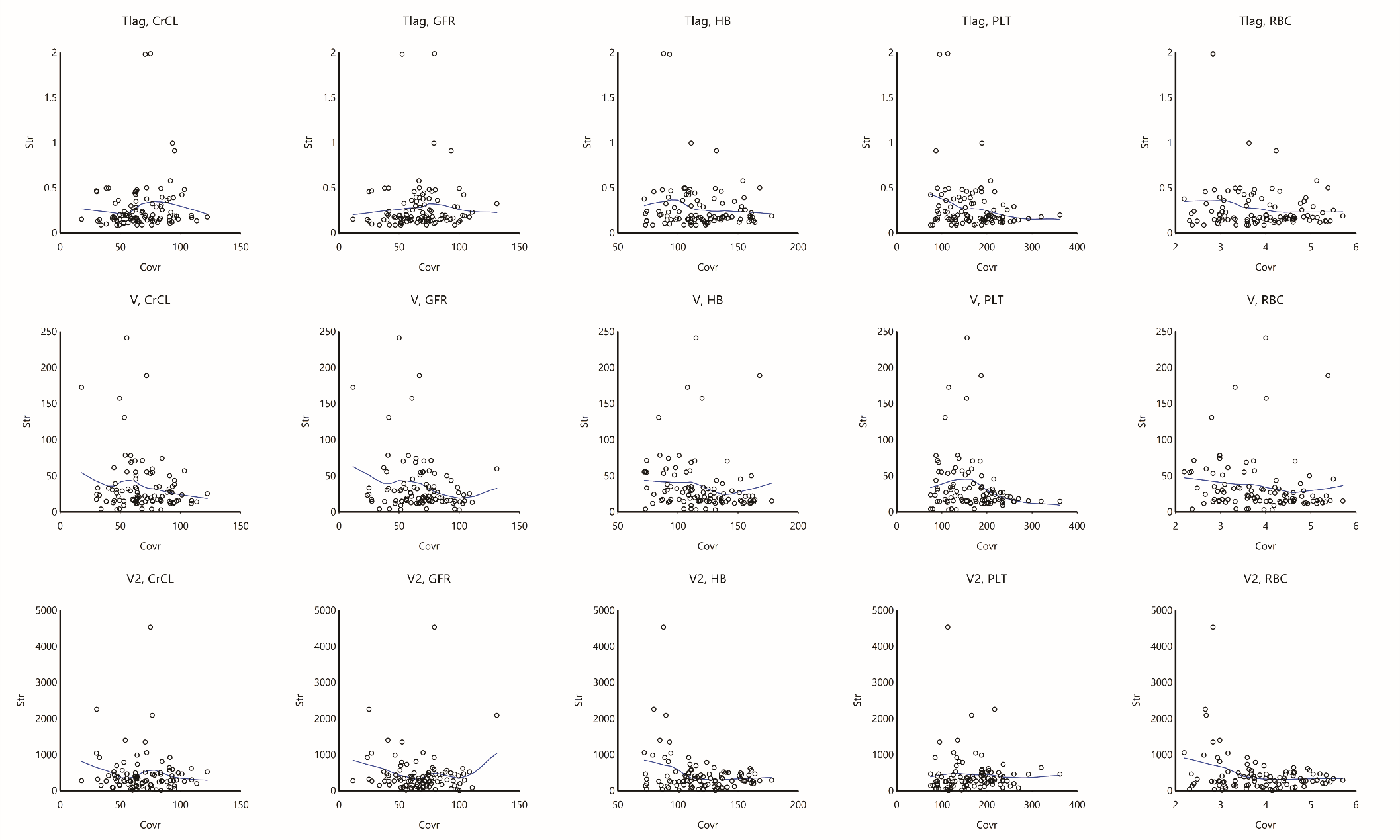

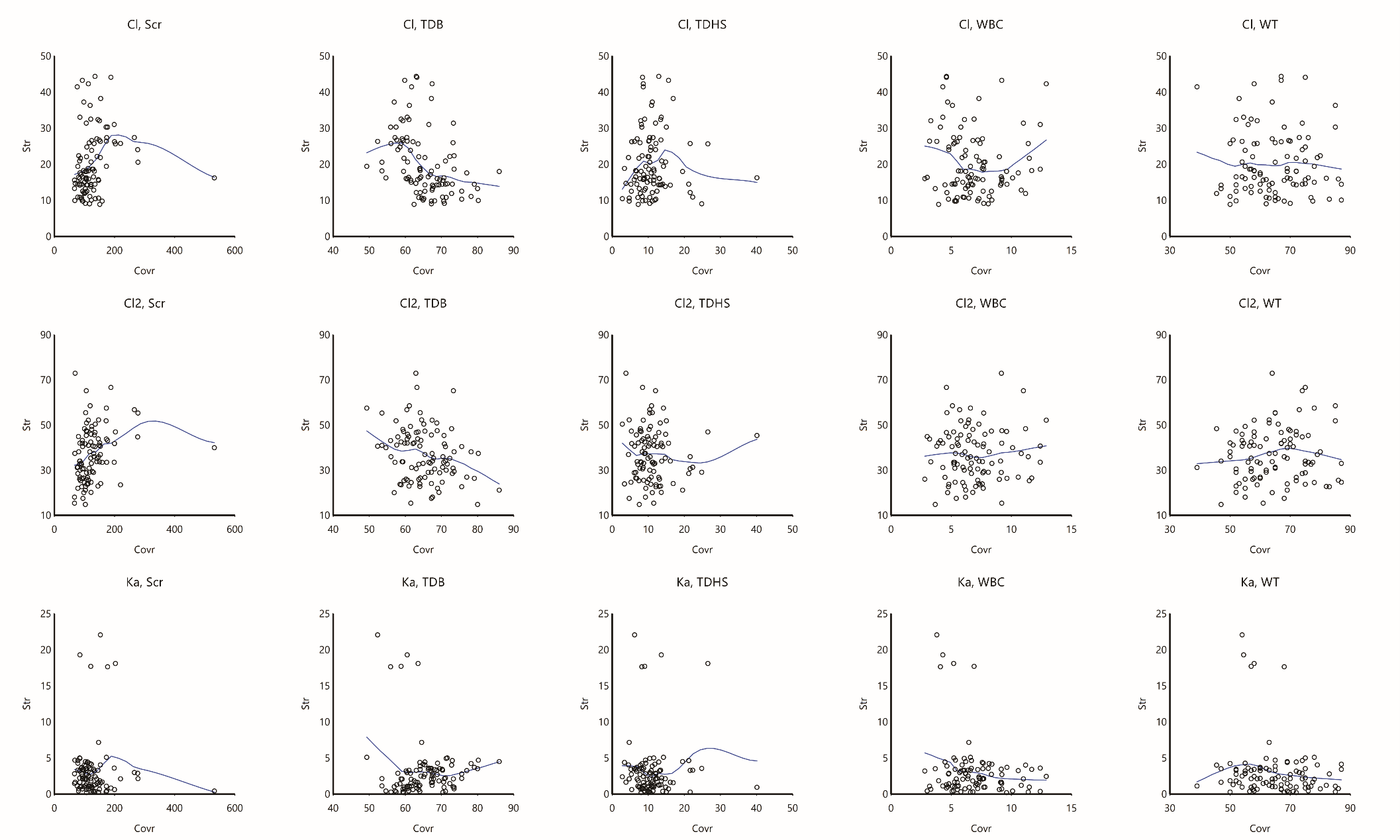

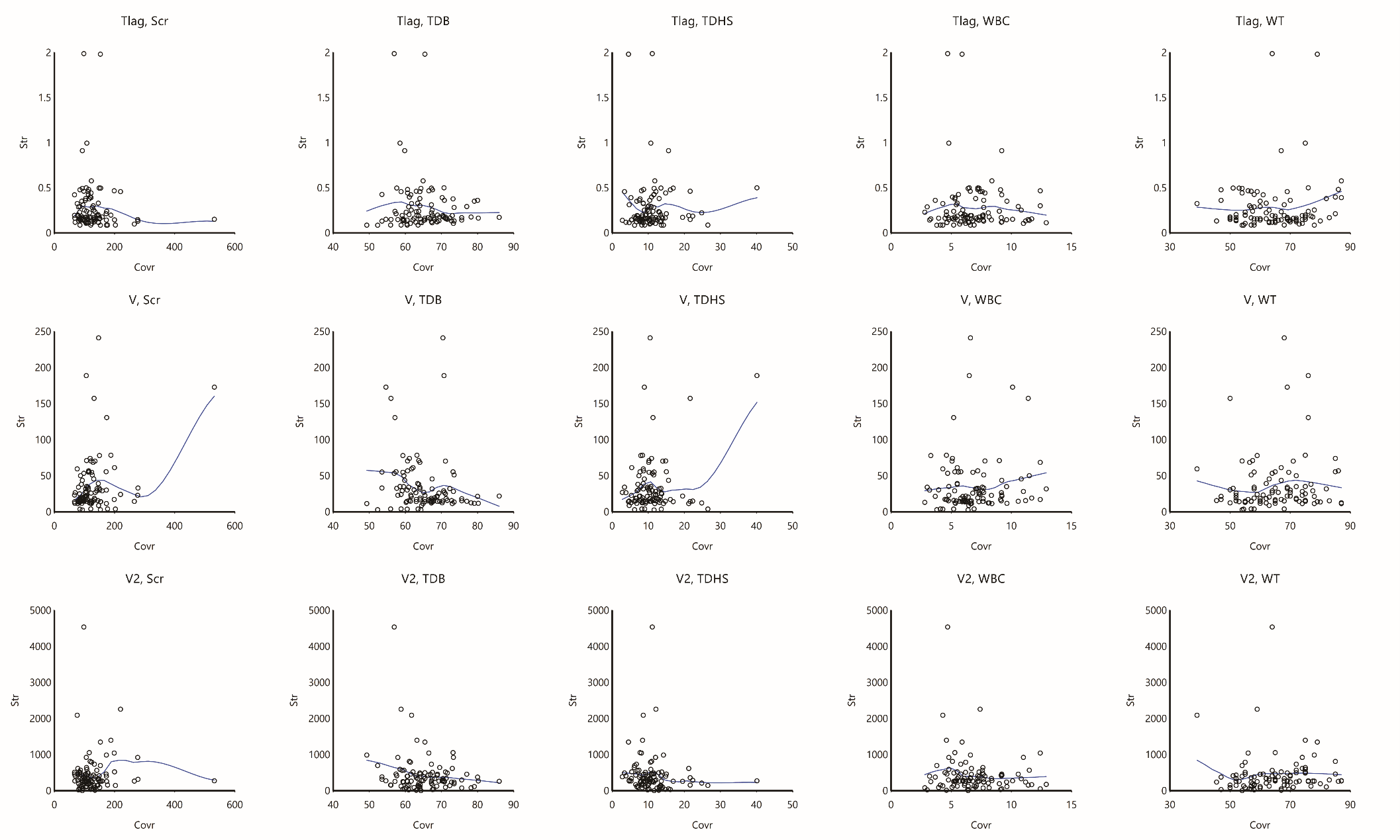

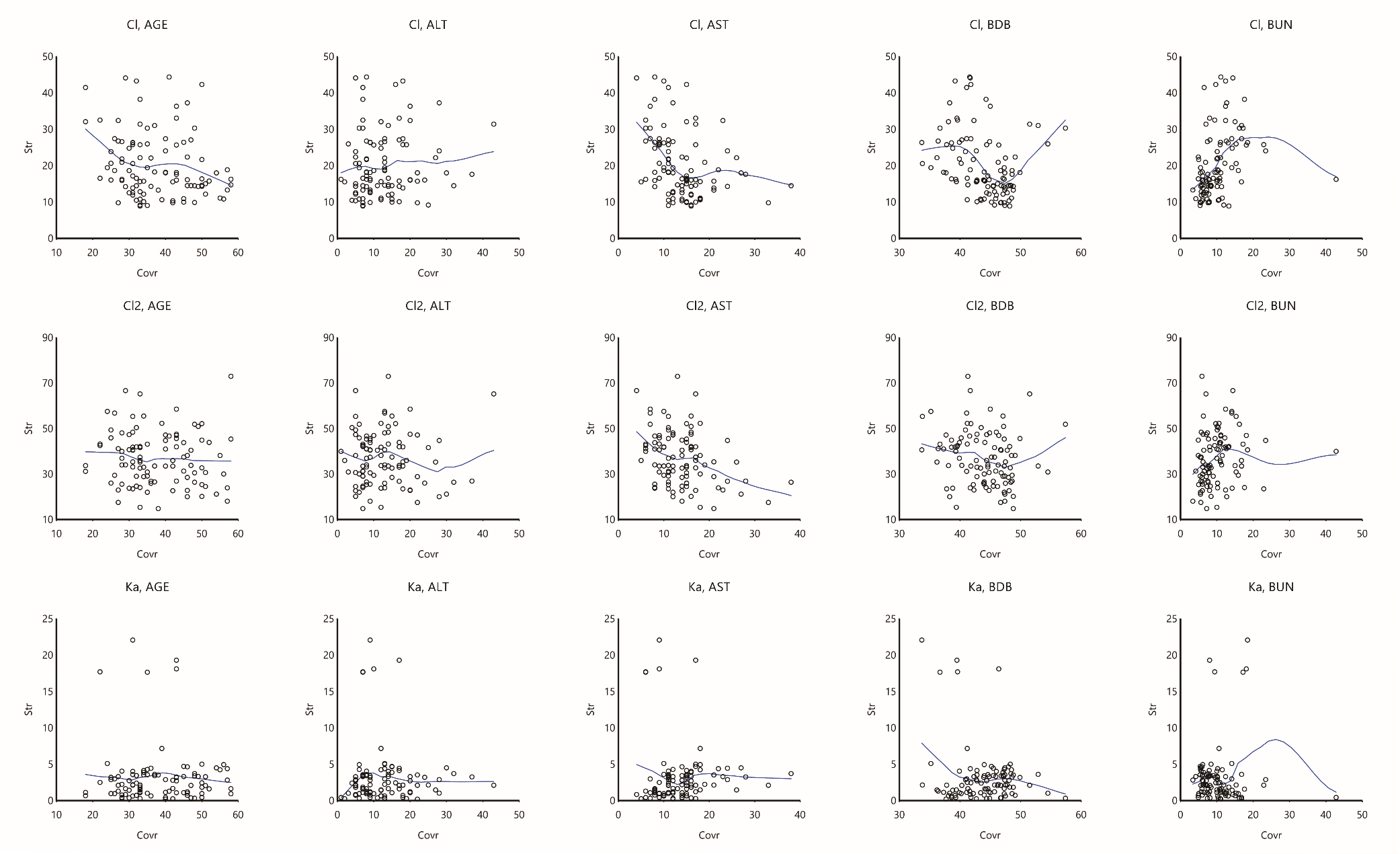

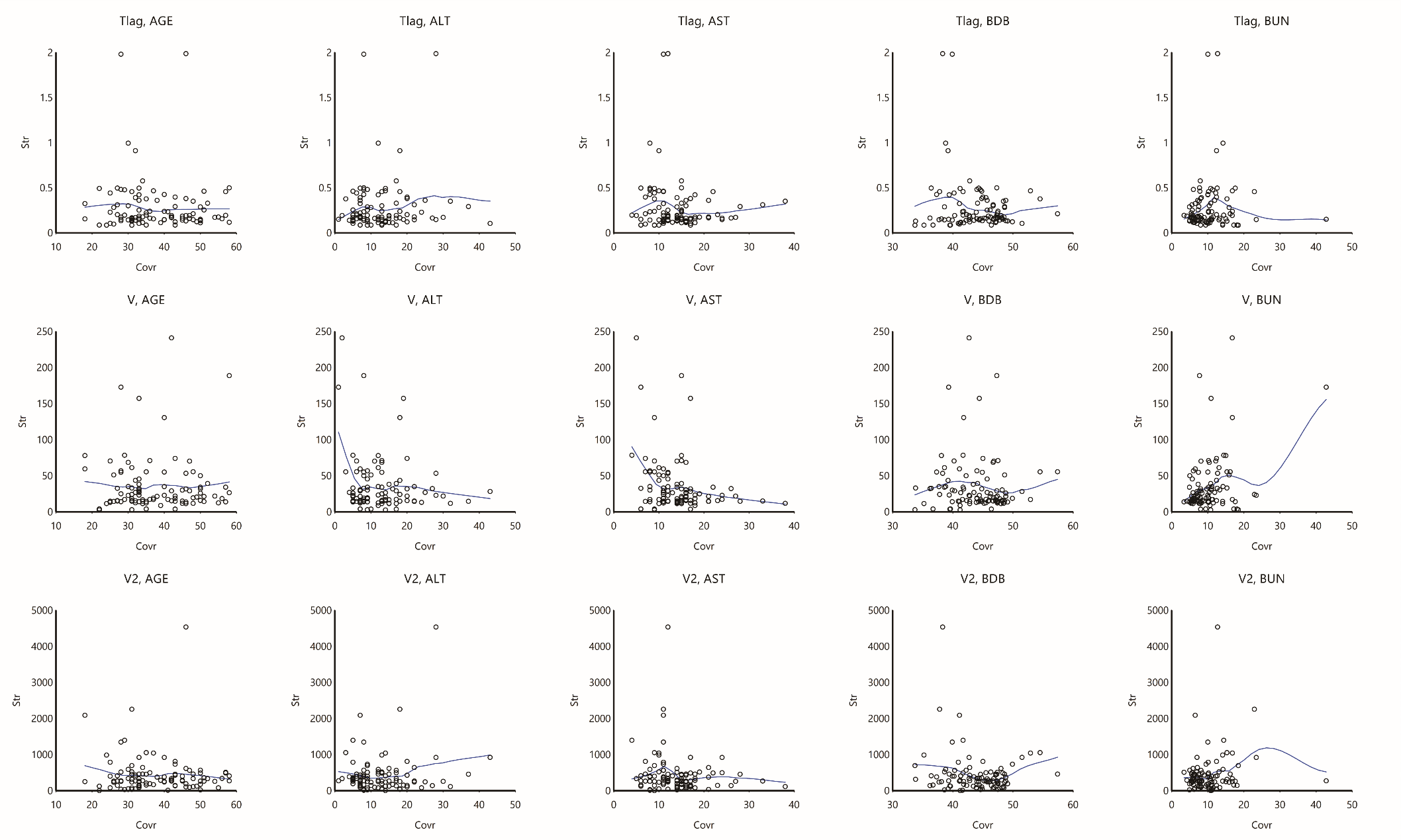


**Figure S2** The correlation plots of covariates vs. PK parameters. Tlag, absorption lag-time; Cl, central clearance; V, central distribution volume; Cl2, intercompartmental clearance (Q); V2, peripheral distribution volume; ka: absorption rate constant; CrCL, creatinine clearance; GFR, glomerular filtration rate; ALT, alanine aminotransferase; AST, aspartate aminotransferase; TDHS, total bilirubin; WBC, white blood cells; HB, erythrocyte; RBC, haemoglobin; PLT, platelet; TDB, serum proteins; BDB, albumin; Scr, serum creatinine; BUN, urea; WT, body weight.

**Supporting text for the EHC process:**

test(){

deriv(A1 = - (A1 * Ke) + (AGI * Ka)- (A1 * K12- A2 * K21)- (A1 * K1b))

urinecpt(A0 = (A1 * Ke))

deriv(AGI = - (AGI * Ka) + (ABile * Kbg))

deriv(A2 = (A1 * K12- A2 * K21))

deriv(ABile = (A1 * K1b)- (ABile * Kbg))

C = A1 / V

dosepoint(A1)

double(flag)

sequence{

flag = 0;

sleep(Tacc);

flag = 1;

sleep(Tsec);

flag = 0;

}

error(CEps = 1)

observe(CObs = C * (1 + CEps))

stparm(V = tvV)

stparm(Ke = tvKe)

stparm(Ka = tvKa)

stparm(K12 = tvK12)

stparm(K21 = tvK21)

stparm(K1b = tvK1b)

stparm(Kbg = flag / tvTsec)

stparm(Tacc = tvTacc)

stparm(Tsec = tvTsec)

fixef(tvV = c(0, 27, ))

fixef(tvCl = c(0, 8, ))

fixef(tvKa = c(0, 3.8, ))

fixef(tvV2 = c(0,520, ))

fixef(tvCl2 = c(0, 11, ))

fixef(tvK1b = c(0, 0.22, ))

fixef(tvTacc = c(0, 5, ))

fixef(tvTsec = c(0, 2.1, ))

ranef(diag(nV, nCl,nKa,nV2,nCl2,nK1b,nTacc,nTsec) = c(1, 1,1,1,1,1,1,1))

}
